# UV_185+254 nm_ photolysis of typical thiol collectors: decomposition efficiency, mineralization and formation of sulfur byproducts

**DOI:** 10.1098/rsos.190123

**Published:** 2019-05-22

**Authors:** Pingfeng Fu, Gen Li, Xiaoting Wu, Xiaofeng Lin, Bolan Lei

**Affiliations:** 1 School of Civil and Resources Engineering, University of Science and Technology Beijing, Beijing 100083, People's Republic of China; 2 Key Laboratory of High-efficient Mining and Safety of Metal Mines, Ministry of Education, Beijing 100083, People's Republic of China

**Keywords:** flotation reagents, thiol collector, UV_185+254 nm_ photolysis, vacuum-UV, mineralization, sulfur byproducts

## Abstract

The decomposition of toxic flotation reagents upon UV_185+254 nm_ irradiation was attractive due to operational simplicity and no dosage of oxidants. In this work, the degradation of typical thiol collectors (potassium ethyl xanthate (PEX), sodium diethyl dithiocarbamate (SDD), *O*-isopropyl-*N*-ethyl thionocarbamate (IET) and dianilino dithiophoshoric acid (DDA)) was investigated by UV_185+254 nm_ photolysis. The degradation efficiencies and mineralization extents of collectors were assessed. The formation of CS_2_ and H_2_S byproducts was studied, and the mechanisms of collector degradation were proposed under UV_185+254 nm_ irradiation. The PEX, SDD and IET were decomposed with nearly 100% removal upon 75 min of UV_185+254 nm_ irradiation. The decomposition rate constants decreased in the order SDD > PEX > IET ≫ DDA, and the DDA was the refractory collector. After 120 min of UV_185+254 nm_ irradiation, 15−45% of carbon and 25−75% of sulfur of collectors were completely mineralized, and the mineralization extent decreased in the order PEX > SDD > IET > DDA. The percentage of gaseous sulfur (CS_2_ and H_2_S) ranged from 0.48 to 4.85% for four collectors, showing the fraction of emitted sulfur byproducts was small. The aqueous CS_2_ concentration increased in the first 10−20 min, and was decreased to a low level of 0.05–0.1 mg l^−1^ at 120 min. Two mechanisms, i.e. direct UV_254 nm_ photolysis and indirect oxidation with free radicals, were responsible for collector decomposition in the UV_185+254 nm_ photolysis.

## 1. Introduction

Froth flotation has become the most widely applied process for separating valuable minerals from ores in mines around the world. Thiol collectors, such as xanthates, dithiophosphates and dithiocarbamates, are important flotation reagents to render sulfide minerals hydrophobic and facilitate bubble attachments [1,2]. To achieve high recovery of non-ferrous metals, the dosage of collectors is frequently ranged from 30 to 300 g ton^−1^ (ore). Therefore, the consumption of thiol collectors becomes very large due to the extremely high amount of treated ores. Even in the 1980s, the global consumption of xanthates had reached above 52 000 tons per year [2]. Nevertheless, nearly 50% of collectors dosed in flotation circuits would be discharged in wastewaters after the mineral flotation [3]. Some collectors and their byproducts are found to be toxic to soil microbes, biota, animals and human beings [4–7]. Previously, the hazards of xanthate to frog embryos have been reviewed [7]. Accordingly, the discharge of collectors from mineral flotation may cause serious environmental pollution. Therefore, it has been of great concern to remove potentially toxic collectors from flotation wastewaters for the sustainable development of mining industry.

Recently, some processes have been developed to remove organic reagents from flotation wastewaters, including adsorption [8], chemical oxidation [9,10], ozonation [11,12] and biodegradation [13,14]. Chemical oxidation and ozonation have exhibited high efficiency in decomposing flotation reagents. But these processes need the dosage of chemical oxidants, such as sodium hypochlorite [9], persulfate [10], hydrogen peroxide [15] and ozone [11]. The usage of highly reactive oxidants in mines makes them less attractive and sometimes expensive. Since most of the mines are located far from industrial zones, there are potential risks associated with long distance transportation and storage of dangerous chemicals. The biodegradation is widely accepted as a low cost process in wastewater treatment. However, previous reports indicate that some flotation reagents are toxic to microbes, considerably reducing microbial activities in the biodegradation of flotation reagents [13,16].

Advanced oxidation processes (AOPs) involving ozone [10], Fenton's reagent [17], hydrogen peroxide [15], persulfate [18,19] and photocatalyst [20–22] have been investigated to effectively degrade flotation collectors, pharmaceuticals and textile dyes. However, most of these studies only concerned the removal efficiencies of xanthates [11,12,15]. Up to now, research on the removal of other thiol collectors such as dithiophosphates and dithiocarbamates has been scarce. Because thiol collectors have different molecular structures [1,23], their decomposition behaviours by the same AOP method may differ. Additionally, the functional groups of all thiol collectors have sulfur atoms [1,23]. So, it is possible to generate toxic sulfur byproducts such as CS_2_ and H_2_S, while organic sulfur of collectors is mineralized to sulfate. For example, the concentrations of aqueous CS_2_ were determined in the ozonation of xanthates [24]. However, the generation of sulfur byproducts receives little concern in the decomposition of thiol collectors by the AOPs [10,12].

The UV-based AOPs, such as UV_254 nm_/O_3_ and UV_254 nm_/TiO_2_ photocatalysis, can effectively decompose flotation collectors and subsequently mineralize various byproducts [11,20]. But these AOPs involve the dosage of oxidants or powder catalysts, making them expensive and complex. The UV_185+254 nm_ photolysis, irradiated by a low-pressure Hg lamp emitting both vacuum-UV (VUV) at 185 nm and UVC at 254 nm, is a simple and promising AOP. The 185 nm VUV irradiation of water can directly generate hydroxyl radicals (OH•), hydrogen radicals (H•), solvated free electrons (e_aq_^−^), superoxide radicals (HO_2_•, O_2_^•−^) and H_2_O_2_ as shown in equations (1.1)−(1.6) [25]. The formation of OH• radicals and superoxide anion (O_2_^•−^) in the VUV irradiation of water have been proven by the electron paramagnetic resonance spectroscopy [26]. The UV_185+254 nm_ photolysis has shown high oxidation capacity for pollutants, such as odour compounds [27], pharmaceuticals [28] and pesticides [29]. Moreover, the operation of UV_185+254 nm_ photolysis is much simpler than the UV_254 nm_/O_3_ and UV_254 nm_/TiO_2_ photocatalysis because no oxidant or catalyst is required. In terms of the practical treatment of flotation wastewaters, the transportation and storage of dangerous oxidants in mines can also be avoided if the UV_185+254 nm_ photolysis is applied. Thus, it is quite necessary to assess the removal performances of flotation reagents by the UV_185+254 nm_ photolysis. However, as far as we know, there is no report on the UV_185+254 nm_ photolysis of organic flotation reagents

1.1 $$H_{2}O+h\nu_{185\mathrm{nm}}\to \mathrm{OH}∙+H^{+}+e_{\mathrm{aq}}^{-},$$

1.2 $$H_{2}O+h\nu_{185\mathrm{nm}}\to \mathrm{OH}∙+H∙,$$

1.3 $$2\mathrm{OH}∙\to H_{2}O_{2},$$

1.4 $$H∙+O_{2}\to HO_{2}∙,$$

1.5 $$HO_{2}∙+H_{2}O\Leftrightarrow H_{3}O^{+}+O_{2}^{∙-}\;\;pK_{a}=4.8$$

1.6 $$\mathrm{and}\;2HO_{2}∙\to O_{2}+H_{2}O_{2}.$$

In this work, the UV_185+254 nm_ photolysis of thiol collectors is investigated by using a 40 W low-pressure Hg lamp, which emits about 10% radiation at 185 nm and 90% radiation at 254 nm. Four thiol collectors, potassium ethyl xanthate (PEX), sodium diethyl dithiocarbamate (SDD), *O*-isopropyl-*N*-ethyl thionocarbamate (IET) and dianilino dithiophoshoric acid (DDA), are selected as typical sulfide mineral collectors. The objectives of this work are (i) to assess the feasibility of decomposing thiol collectors by the UV_185+254 nm_ photolysis, (ii) to determine the generation of CS_2_ and H_2_S byproducts, as well as (iii) to propose the collector decomposition mechanisms under UV_185+254 nm_ irradiation. The mineralization of collectors is examined by measuring total organic carbon (TOC) and SO_4_^2−^ concentrations. The amounts of gaseous CS_2_ and H_2_S, as well as the concentration of aqueous CS_2_, were measured to study the generation of sulfur byproducts.

## 2. Material and methods

### 2.1. UV lamps and chemicals

Two types of low-pressure Hg lamps with electrical power of 40 W were purchased from Bright Star Light & Electricity Co., Ltd, Guangdong, China. The UV_185+254 nm_ lamp transmitted both 185 nm VUV and 254 nm UV light through high-purity quartz glass, but the UV_254 nm_ lamp only transmitted 254 nm UV light. The UV_254 nm_ irradiation intensity of UV_185+254 nm_ lamp was about 90% of UV_254 nm_ lamp. The lamps had a length of 1199 mm and a diameter of 19 mm.

The PEX and SDD with analytical grade were purchased from Aladdin Chemical Reagent Co., Ltd, Shanghai, China. The industrial grade IET and DDA were purchased from Tieling Flotation Reagents Co., Ltd, Liaoning, China. Their molecular structures, molecular formulas and abbreviations are summarized in table 1. Other chemicals such as cupric acetate, lead acetate, diethylamine, triethanolamine, silver sulfate and mercury sulfate were of analytical grade and purchased from Sinopharm Chemical Reagent Co., Ltd, Beijing, China. Deionized water was used in the degradation experiments.

**Table 1.** Collector name, molecular structure, molecular formula and abbreviation.

collector name	molecular structure	molecular formula	abbreviation
potassium ethyl xanthate	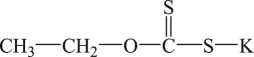	C_2_H_4_OCSSK	PEX
sodium diethyl dithiocarbamate	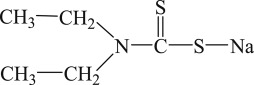	(C_2_H_5_)_2_NCSSNa	SDD
*O*-isopropyl-*N*-ethyl thionocarbamate	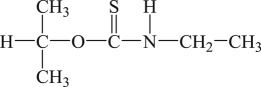	(CH_3_)_2_CHOCSNHC_2_H_5_	IET
dianilino dithiophoshoric acid	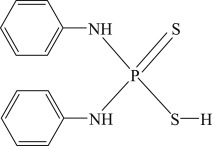	(C_6_H_5_NH)_2_PSSH	DDA

### 2.2. Experimental set-up and degradation procedures

**Figure 1.**
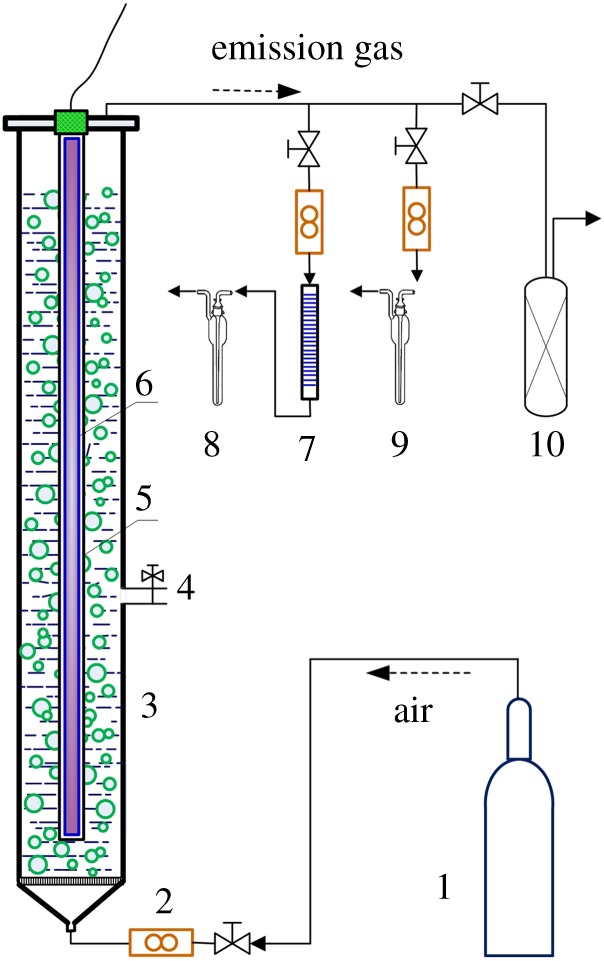
Schematic diagram of experimental set-up. (1, air bottle; 2, flow meter; 3, purged reactor; 4, sampling valve; 5, quartz tube; 6, UV_185+254 nm_ or UV_254 nm_ lamp; 7, absorbent cotton with lead acetate; 8, CS_2_ absorption liquid; 9, H_2_S absorption liquid; 10, activated carbon column.)

All degradation experiments were conducted with a batch mode in a jacket glass reactor connected to a thermostatic bath. The schematic diagram of the experimental set-up is shown in figure 1. The cylindrical reactor, with 1240 mm height and 53 mm internal diameter, was installed at its axis with a quartz tube with a height of 1220 mm and outer diameter of 21 mm. The UV_185+254 nm_ or UV_254 nm_ lamp was inserted into the quartz tube. The optical distance from tube surface to the internal surface of the reactor was 16 mm. The air was continuously purged into the reactor through a porous glass plate with a flow rate of 1.67 l min^−1^. The injected air stream not only provided dissolved oxygen for degradation reactions, but also stirred the collector solutions. The degradation experiments were conducted at 25 ± 2°C.

Prior to photolysis experiments, the collector (0.2 g) was dissolved in 2 l deionized water to prepare a PEX (SDD, IET or DDA) solution of 100 mg l^−1^ concentration. The initial pH was adjusted to 7.0−12.0 using 0.05 mol l^−1^ NaOH or HCl solution. When the collector solution was introduced into the reactor purged with an air stream, the UV_185+254 nm_ or UV_254 nm_ lamp was turned on to carry out photolysis experiments. The irradiation time was controlled to be 120 min. Because CS_2_, H_2_S and other sulfur byproducts were emitted into gas phase, the emission gas was introduced into an activated carbon column to remove toxic byproducts. The aqueous samples were taken at designated intervals to determine the concentrations of collector, chemical oxygen demand (COD), TOC, CS_2_ and SO_4_^2−^ ions.

### 2.3. Analysis and calculation

#### 2.3.1. Determination of collector concentration, COD and TOC

The concentration of thiol collectors (PEX, SDD, IET and DDA) was determined by a UV–vis spectroscopic method [10,11,13,30]. The absorbance of collector solution was recorded by a UV–vis spectrophotometer (UV-5500PC, Shanghai Metash Instruments Co. Ltd, China). The COD was determined by the standard dichromate method (HJ/T 399-2007). The TOC of water samples was measured by using a Shimadzu TOC-V organic carbon analyzer. The carbon mineralization extent (*γ*_c_) of the collector (PEX, SDD, IET and DDA) was calculated as the below equation

2.1 $$\gamma_{c}=\frac{\mathrm{TO}C_{0}-\mathrm{TO}C_{t}}{\mathrm{TO}C_{0}}\times 100\text{\%},$$

where TOC_0_ and TOC*_t_* (mg l^−1^) were the TOC concentration at initial and time *t*, respectively.

#### 2.3.2. Determination of concentrations of aqueous CS_2_ and SO_4_^2−^ ions

The aqueous CS_2_ concentration was measured by a diethylamine cupric acetate spectrophotometric method (GB/T 15504-1995). Fifty millilitres of water sample were continuously purged by a 100 ml min^−1^ N_2_ stream for 1 h to volatilize CS_2_, which was absorbed by a diethylamine and cupric acetate mixed liquid. Then the absorbance of absorption liquid was measured at 430 nm using dehydrated alcohol as a reference solution. The concentration of sulfate was determined by a barium chromate spectrophotometry method (HJ/T 342-2007). Because SO_4_^2−^ ions, with the highest valence of sulfur, were the final oxidization product of organic sulfur in collectors, the sulfur mineralization extent (*γ*_s_) of the collector was defined as the below equation [31]

2.2 $$\gamma_{s}=\frac{M}{n\times 96}\times \frac{C_{{\mathrm{SO}}_{4}^{2-},t}}{C_{0}}\times 100\text{\%}$$

where *M* and *n* were the molecular weight and number of sulfur atom in the collector (PEX, SDD, IET or DDA), respectively, $C_{{\mathrm{SO}}_{4}^{2-},t}$ (mg l^−1^) was the concentration of SO_4_^2−^ ions at time *t*, and *C*_0_ (mg l^−1^) was the initial concentration of the collector.

#### 2.3.3. Measurement of the amount of gaseous CS_2_ and H_2_S

The amount of gaseous CS_2_ emitted from the reactor for 120 min was measured by a diethylamine spectrophotometric method (GB/T 14680-93). By adding 5.0 mg cupric acetate, 2.5 ml diethylamine and 2.5 ml triethanolamine into a 500 ml volumetric flask, the CS_2_ absorption liquid was prepared by diluting the mixture with dehydrated alcohol to 500 ml. As shown in figure 1, before the emission gas was introduced into CS_2_ absorption liquid, it was first passed through a glass tube filled by absorbent cotton coated with lead acetate to remove H_2_S. The amount of gaseous H_2_S for 120 min was measured by a methylene blue spectrophotometric method (GB/T 11742-89). By adding 4.3 g cadmium sulfate, 0.30 g NaOH and 10.0 g polyvinyl alcohol ammonium phosphate into a 1000 ml volumetric flask, the H_2_S absorption liquid was prepared by diluting the mixture with deionized water to 1000 ml.

## 3. Results and discussion

### 3.1. Effect of UV wavelength

**Figure 2.**
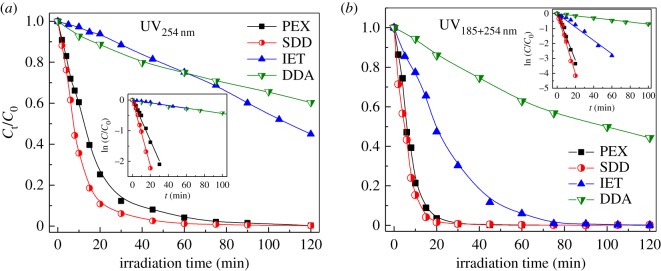
The variations of relative concentration (*C*_t_/*C*_0_) with irradiation time upon UV_254 nm_ (*a*) and UV_185+254 nm_ (*b*) irradiation. The insets are the pseudo-first-order kinetic fitting of ln(*C*_t_/*C*_0_) versus time *t*. Experimental conditions: collector concentration of 100 mg l^−1^ and initial pH of 10.0.

The photolysis of thiol collectors (PEX, SDD, IET and DDA) upon UV_254 nm_ or UV_185+254 nm_ irradiation is shown in figure 2. The removal efficiency of collectors and decomposition rate constants are summarized in table 2. In this work, overall kinetics of the collector degradation can be described by a simple pseudo-first-order rate law [32]. As shown in figure 2*a* and table 2, the removal efficiency of PEX and SDD at 75 min reached 97.93% and 99.16%, respectively, which was much higher than that (about 30%) of IET and DDA upon UV_254 nm_ irradiation. In UV-irradiated solutions, the decomposition of pollutants is frequently initiated by excited molecules with absorbing UV irradiation [33]. Among four collectors, the PEX and SDD may have higher mole absorptivity of UV_254 nm_ irradiation than IET and DDA, resulting in higher removal efficiencies. In most mines, residual flotation reagents are usually removed in tailing ponds by a natural degradation process involving sunlight and oxygen. As given in table 2, the half-life of IET and DDA was almost one order of magnitude larger than that of PEX and SDD. Therefore, it can reasonably be inferred that after the natural degradation, residual IET and DDA in tailing ponds would have higher concentrations than the PEX and SDD.

**Table 2.** The removal efficiencies of collectors, decomposition rate constants (*k*_collector_), half-lives (*t*_1/2_) and correlation coefficients (*R*^2^) in the degradation of thiol collectors under UV_185+254 nm_ and UV_254 nm_ irradiation, respectively.

collectors	UV irradiation	removal efficiency (%)		*k*_collector_ (min^−1^)	*t*_1/2_ (min)	*R*^2^
		20 min	75 min			
PEX	UV_185+254 nm_	96.51	99.86	0.1565	4.43	0.982
	UV_254 nm_	74.77	97.93	0.06629	10.46	0.989
SDD	UV_185+254 nm_	98.45	99.88	0.2005	3.02	0.990
	UV_254 nm_	89.21	99.16	0.1076	6.44	0.992
IET	UV_185+254 nm_	52.62	99.08	0.04485	15.45	0.986
	UV_254 nm_	6.25	31.20	0.00447	155.07	0.988
DDA	UV_185+254 nm_	13.98	43.26	0.00701	98.88	0.998
	UV_254 nm_	11.31	29.17	0.00438	158.25	0.991

As exhibited in figure 2*b* and table 2, the removal efficiency of PEX and SDD had reached above 96% even at 20 min under UV_185+254 nm_ irradiation. At 75 min, nearly 100% removal of PEX, SDD and IET was achieved, revealing that most thiol collectors could be effectively degraded by the UV_185+254 nm_ photolysis without adding any oxidant. The decomposition rate constant (*k*_collector_) of collectors upon UV_185+254 nm_ irradiation decreased in the order *k*_SDD_ (0.2005 min^−1^) > *k*_PEX_ (0.1565 min^−1^) > *k*_IET_ (0.04485 min^−1^) ≫ *k*_DDA_ (0.00701 min^−1^). As listed in table 2, the *k*_collector_ values for four collectors under UV_185+254 nm_ irradiation were 1.60−10.03 times higher than those achieved upon UV_254 nm_ irradiation, showing the enhanced degradation of thiol collectors by the UV_185+254 nm_ photolysis. However, the DDA, with very low *k*_collector_ value, can be considered as a refractory collector for the UV_185+254 nm_ photolysis.

### 3.2. Effect of initial pH

**Figure 3.**
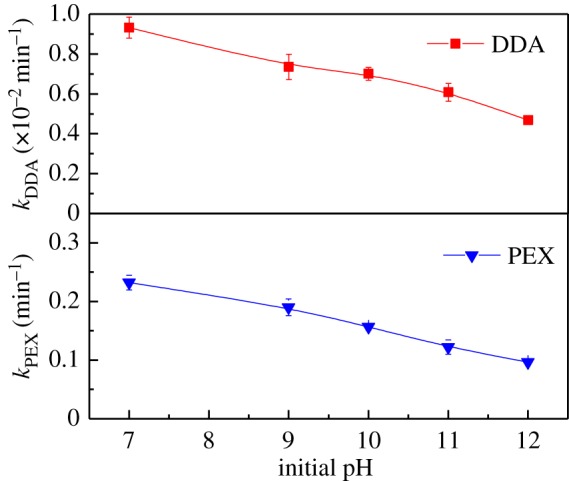
The variations of the *k*_PEX_ and *k*_DDA_ with initial pH under UV_185+254 nm_ irradiation. Experimental conditions: collector concentration of 100 mg l^−1^.

The flotation of sulfide minerals is usually conducted in alkaline pulps. Thus, residual collectors are present in the flotation wastewaters at pH range from neutral to alkaline [34]. In this work, the effect of initial pH on the UV_185+254 nm_ photolysis of PEX and DDA was investigated. As shown in figure 3, both the *k*_PEX_ and *k*_DDA_ values gradually decreased as initial pH rose. The decomposition rate constants obtained at pH 7.0 were 2.4 times for PEX and 4.9 folds for DDA higher than those achieved at pH 12.0, respectively. It suggests that the neutral pH can facilitate the decomposition of flotation collectors upon UV_185+254 nm_ irradiation. In the previous studies, the higher decomposition efficiencies were also achieved at lower pH during the UV_185+254 nm_ photolysis of 1,4-dioxane [30] and 4-*tert*-octylphenol [35].

Upon 185 nm VUV irradiation, the homolysis and photochemical ionization of H_2_O occur with the generation of OH• radicals (equations (1.1) and (1.2)), and OH• radicals (or HO_2_•) can recombine to form H_2_O_2_ (equations (1.3) and (1.6)) [25]. Under UV_185+254 nm_ irradiation, H_2_O_2_ can be decomposed by absorbing UV_254 nm_ light to form OH• radicals (equation (3.1)). Accordingly, OH• radicals and H_2_O_2_ are in equilibrium under UV irradiation offered by the UV_185+254 nm_ lamp. On the other hand, the concentration of H_2_O_2_ is also dependent on the pH value of the reaction system. As given in equation (3.2), H_2_O_2_ itself is in equilibrium with OH^−^ anions [36]. Additionally, HO_2_• radicals are also in equilibrium with protons ((equation (1.5)). According to equations (1.5), (1.6) and (3.2), it can be seen that high H^+^ concentration (i.e. low pH) promotes the equilibrium to form H_2_O_2_. In turn, increased concentration of H_2_O_2_ results in the formation of more OH• radicals (equation (3.1)). This equilibrium consideration may explain why the *k*_PEX_ and *k*_DDA_ values were higher at the neutral pH under UV_185+254 nm_ irradiation

3.1 $$H_{2}O_{2}+h\nu_{254\;\mathrm{nm}}\to \mathrm{OH}∙+\mathrm{OH}∙$$

and

3.2 $$H_{2}O_{2}+OH^{-}\Leftrightarrow H_{2}O+HO_{2}^{-}\;\;\;pK_{a}=11.62.$$

In addition, the equilibrium of carbonate may also influence the pH dependence of the collector decomposition. The mineralization of contaminants by UV-based AOPs results in the formation of CO_2_, subsequently increasing the aqueous concentration of CO_3_^2−^ anions. According to the equilibrium of CO_3_^−^ in water, HCO_3_^−^ and CO_3_^2−^ anions dominate the equilibrium approximately at neutral to weak basic pH (6 < pH < 10) and at strong basic pH (pH > 10), respectively. These carbonate species can scavenge OH• radicals. However, CO_3_^2−^ anions have a larger scavenging capacity than HCO_3_^−^ [37]. Thus, at higher initial pH, more OH• radicals would be scavenged by CO_3_^2−^ anions, resulting in lower decomposition rate constants of PEX and DDA.

### 3.3. The mineralization of thiol collectors

**Figure 4.**
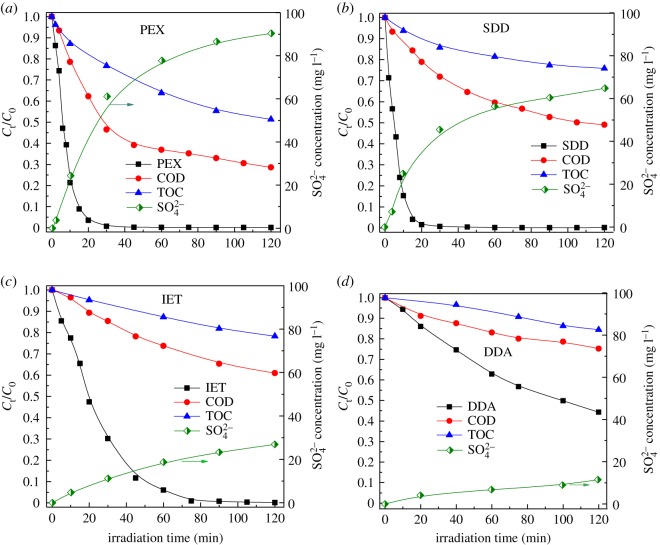
The variations of SO_4_^2−^ concentration and relative concentrations of collector (PEX (*a*), SDD (*b*), IET (*c*) and DDA (*d*)), COD and TOC with UV_185+254 nm_ irradiation time. Experimental conditions: collector concentration of 100 mg l^−1^ and initial pH of 10.0.

To investigate the mineralization of thiol collectors under UV_185+254 nm_ irradiation, the concentrations of COD, TOC and SO_4_^2−^ ions were measured with the results shown in figure 4. For each collector, the relative concentrations of COD and TOC declined, and the SO_4_^2−^ concentration increased as the collector was decomposed. It suggested that the byproducts had been further degraded with the formation of CO_2_ and SO_4_^2−^ ions under UV_185+254 nm_ irradiation. As indicated in figure 4, the TOC was reduced just a bit while the evident decrease in COD was observed for each collector. Thus, the removal efficiency of COD was higher than the mineralization extent of carbon as given in table 3. By comparing the decomposition rate constants for collector and COD removal, as summarized in tables 2 and 3, the *k*_collecor_ for each collector was 3−21 folds higher than the *k*_COD_ value. It clearly indicated that only a small fraction of organic carbon in collectors was mineralized to CO_2_ upon UV_185+254 nm_ irradiation although nearly 100% removal of PEX, SDD and IET was achieved. For four collectors, the mineralization extent of carbon (*γ*_c_) at 120 min increased in the order DDA < IET < SDD < PEX. In particular, the *γ*_c_ for the PEX was much higher than that for SDD, IET and DDA, indicating that the byproducts derived from PEX seemed to be readily decomposed by OH• radicals.

**Table 3.** The removal efficiencies of COD, decomposition rate constants for COD removal (*k*_COD_), correlation coefficients (*R*^2^) and the mineralization extents of carbon and sulfur in the UV_185+254 nm_ photolysis of thiol collectors.

collector	removal of COD			mineralization extent (%)	
	removal efficiency of COD (%)	*k*_COD_ (min^−1^)	*R*^2^	carbon	sulfur
PEX	71.40	0.02245	0.9896	48.62	74.83
SDD	50.92	0.00953	0.9844	24.17	57.69
IET	39.04	0.00455	0.9878	21.68	41.19
DDA	24.77	0.00259	0.9783	15.56	16.75

As shown in figure 4, the concentration of SO_4_^2−^ ions increased up to 90.36 mg l^−1^ for PEX, 64.79 mg l^−1^ for SDD, 26.90 mg l^−1^ for IET and 11.48 mg l^−1^ for DDA, respectively, under UV_185+254 nm_ irradiation for 120 min. The SO_4_^2−^ ions were the final sulfur product in the oxidation of organic sulfur in thiol collectors [11,12,24]. The occurrence of SO_4_^2−^ ions with increased concentrations well demonstrated that organic sulfur of collectors could be completely mineralized [24]. As summarized in table 3, the mineralization extent of sulfur (*γ*_s_) at 120 min had reached 74.83% for PEX, 57.69% for SDD, 41.19% for IET and 16.75% for DDA, respectively. Thiol collectors except for DDA had much higher *γ*_s_ values than the *γ*_c_, suggesting that the conversion of organic sulfur to SO_4_^2−^ was more efficient than the mineralization of organic carbon to CO_2_. By considering *γ*_c_ and *γ*_s_ together, the mineralization extent of thiol collectors decreased in the order PEX > SDD > IET > DDA upon UV_185+254 nm_ irradiation. In particular, the PEX and SDD had much larger extent of mineralization when compared with IET and DDA at the same degradation conditions.

### 3.4. Generation of CS_2_ and H_2_S byproducts

Sulfur byproducts, such as carbon disulfide (CS_2_), carbonyl sulfide (COS), hydrogen sulfide (H_2_S), sulfite (SO_3_^2−^) and thiosulfate (S_2_O_3_^2−^), were detected in the oxidization of xanthates by the AOPs [15,24,38–40]. Among these sulfur byproducts, the CS_2_, COS and H_2_S are toxic and highly volatile contaminants [41]. For example, CS_2_ is considered as a hazard air pollutant under the Title III of the 1990 Clean Air Act Amendment (CAAA) of the USA [42]. Accordingly, while toxic sulfur byproducts are emitted from air purged flotation wastewaters into the atmosphere or indoor environment, it may pose a significant hazard to safety, health and the environment (SHE). However, to the best of our knowledge, no work is available in the literature on the quantitative determination of emitted CS_2_ and H_2_S in gas phase when thiol collectors are decomposed by the AOPs. For the aqueous CS_2_ byproduct, Fu *et al*. [11,24] had measured the CS_2_ concentration while degrading xanthates by the O_3_ and UV/O_3_ processes. However, the evolutions of aqueous CS_2_ byproduct has not yet been investigated during the degradation of thiol collectors, except for xanthates by the AOPs.

**Figure 5.**
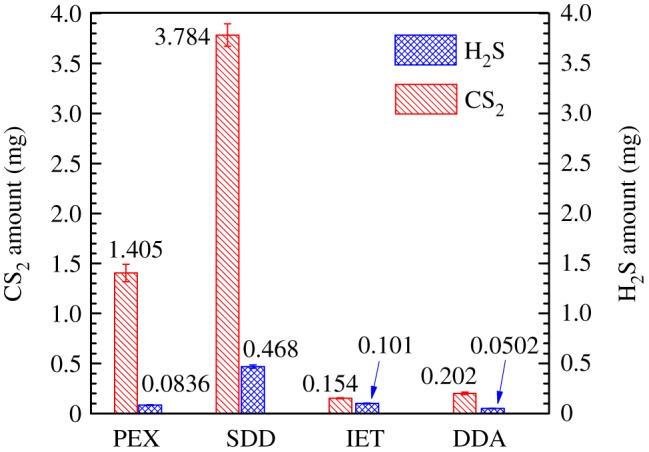
The amounts of emitted CS_2_ and H_2_S in gas phase by the UV_185+254 nm_ photolysis of thiol collectors for 120 min. Experimental conditions: collector concentration of 100 mg l^−1^ and initial pH of 10.0.

In this work, the amounts of gaseous CS_2_ and H_2_S emitted from air purged solutions were measured using the CS_2_ and H_2_S absorption liquids, respectively. As illustrated in figure 5, upon UV_185+254 nm_ irradiation of 100 mg l^−1^ collector solutions for 120 min, the amount of gaseous CS_2_ reached 1.405 mg for PEX, 3.784 mg for SDD, 0.154 mg for IET and 0.202 mg for DDA, respectively. The results indicated that the SDD and PEX released much larger amount of gaseous CS_2_ than IET and DDA. For the emission of H_2_S byproduct, gaseous H_2_S reached 0.0836 mg for PEX, 0.468 mg for SDD, 0.101 mg for IET and 0.0502 mg for DDA, respectively. Among four collectors, the SDD had released the largest amount of H_2_S into gas phase. For each collector, the amount of gaseous CS_2_ released was larger than that of H_2_S, and the trends became more evident for PEX and SDD. By considering CS_2_ and H_2_S together, the amount of gaseous sulfur byproducts released upon UV_185+254 nm_ irradiation increased in the order IET ≈ DDA < PEX < SDD. According to these results, it could be seen that the release of gaseous sulfur byproducts was quite diverse due to their different molecular structures and sulfur contents of thiol collectors.

As mentioned above, the emitted CS_2_ and H_2_S are toxic gases. Thus, it is essential to indicate the percentage of gaseous sulfur to total sulfur in collectors. By assuming that no volatile sulfur byproducts except for CS_2_ and H_2_S are present in emission gas, the percentage of gaseous sulfur (*β*_s,g_) can be defined according to the below equation

3.3 $$\beta_{s,g}=\frac{m_{1}\times (64/76)+m_{2}\times (32/34)}{((n\times 32)/M)\times C_{0}\times V}\times 100\text{\%},$$

where *m*_1_ and *m*_2_ (mg) were the amount of emitted CS_2_ and H_2_S into gas phase for 120 min, *n* was the number of S atom in collectors and *V* (l) was the volume of collector solutions. Thus, the *β*_s,g_ values for four collectors are summarized in table 4. Except for the SDD, the *β*_s,g_ for PEX, IET and DDA was very low, showing that only a small fraction of sulfur in thiol collector was released into emission gas in the UV_185+254 nm_ photolysis. However, a previous study had estimated that 20.6% of total sulfur in *n*-butyl xanthate was released into gas phase after the oxidation by O_3_ [12]. These data were achieved by subtracting total sulfur in *n*-butyl xanthate with sulfur in SO_4_^2−^ ions. Since other sulfur byproducts such as sulfite, thiosulfate as well as organic sulfur compounds would be also presented in aqueous solutions, 20.6% of sulfur released in gas phase might be overestimated by Yan *et al.* [12].

**Table 4.** The percentage of gaseous sulfur (*β*_s,g_) in the UV_185+254 nm_ photolysis of thiol collectors for 120 min.

collector	PEX	SDD	IET	DDA
*β*_s,g_	1.57	4.85	0.53	0.48

**Figure 6.**
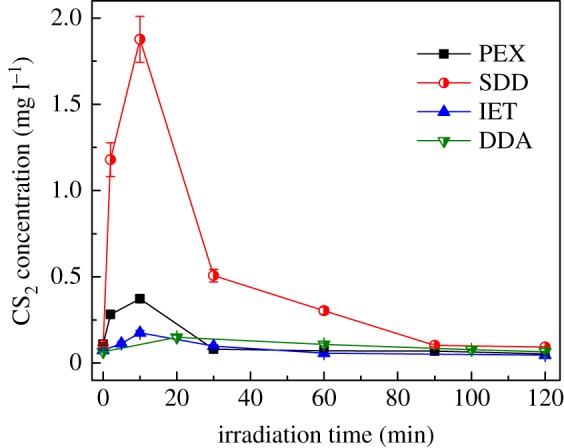
The variations of aqueous CS_2_ concentration with UV_185+254 nm_ irradiation time. Experimental conditions: collector concentration of 100 mg l^−1^ and initial pH of 10.0.

In this work, aqueous CS_2_ concentrations were also measured. As presented in figure 6, the CS_2_ concentration for each collector rapidly increased to its maximum value in the first 10−20 min, and then gradually decreased to a low level under UV_185+254 nm_ irradiation. The measured maximum concentration of CS_2_ was 0.37 mg l^−1^ for PEX, 1.88 mg l^−1^ for SDD, 0.18 mg l^−1^ for IET and 0.15 mg l^−1^ for DDA, respectively. It can be clearly seen that the decomposition of SDD can generate much more CS_2_ in comparison to other collectors. Additionally, the order of maximum CS_2_ concentration for four collectors was well consistent with that for the amount of gaseous CS_2_ as shown in figure 5. As shown in figure 6, residual CS_2_ concentrations for all collectors were decreased to a low level of 0.05−0.1 mg l^−1^ after 120 min of UV_185+254 nm_ irradiation. By considering the remarkable increase in SO_4_^2−^ concentrations shown in figure 4, it can be reasonably inferred that most of CS_2_ byproduct was converted to SO_4_^2−^ ions by OH• radicals with the pathways as elucidated in our previous work [24].

As discussed above, the decomposition of SDD and PEX had generated a larger amount of CS_2_ in comparison to IET and DDA. As summarized in table 1, both the sulfur content and molecular structure of thiol collectors are quite different from each other. Accordingly, the different sulfur contents and decomposition mechanisms caused by different molecular structures would be associated with the formation of sulfur byproducts under UV_185+254 nm_ irradiation. The sulfur contents of PEX, SDD, IET and DDA are 40.25%, 37.42%, 21.77% and 22.85%, respectively. The high sulfur contents of PEX and SDD may well elucidate their large amount of generated CS_2_ byproduct.

Additionally, the generation mechanisms of CS_2_ from thiol collectors might also significantly influence its amount. However, up to now, the reports on the decomposition mechanisms of thiol collectors by the AOPs are scare. For the ozonation of xanthates, it is suggested that the attack on the C−O bond of xanthates by OH• radicals and nucleophilic reactions occurring for the C=S bond of the −CSS^−^ group could result in the generation of the CS_2_ [24,39]. In this case, the C−O bond in the PEX and C−N bond in the SDD have high electron density, which may be preferentially attacked by OH• radicals. Thus, the CS_2_ may be generated from the PEX and SDD under UV_185+254 nm_ irradiation according to equations (3.4) and (3.5), respectively. As presented in table 1, the IET and DDA had molecular structures that were different to the PEX and SDD. The pathways of CS_2_ generated from IET and DDA may be different from that for the PEX and SDD. In our future work, attention should be paid to elucidating the generation pathways of sulfur byproducts

3.4 $$CH_{3}CH_{2}\mathrm{OCSSK}+\mathrm{OH}∙+1/2O_{2}\to CH_{3}CH_{2}\mathrm{OH}+CS_{2}+K^{+}+O_{2}^{∙-}$$

and

3.5 $${\left(CH_{3}CH_{2}\right)}_{2}\mathrm{NCSSNa}+\mathrm{OH}∙+1/2O_{2}\to {\left(CH_{3}CH_{2}\right)}_{2}\mathrm{NH}+CS_{2}+K^{+}+O_{2}^{∙-}$$

### 3.5. The mechanisms of UV_185+254 nm_ photolysis of thiol collectors

**Figure 7.**
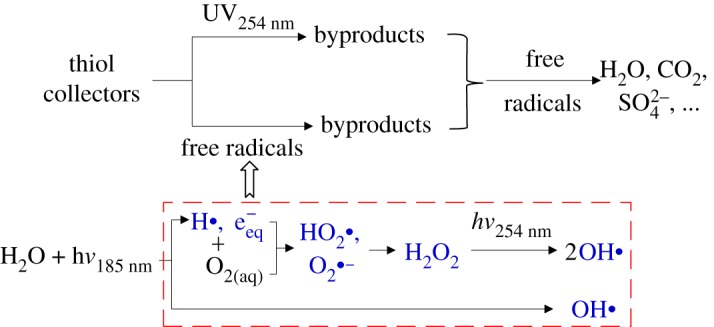
Proposed degradation mechanisms of thiol collectors under UV_185+254 nm_ irradiation.

As shown in figure 2 and table 2, thiol collectors could be degraded by both UV_254 nm_ and UV_185+254 nm_ photolysis, but with higher degradation rate constants under UV_185+254 nm_ irradiation. As mentioned above, free radicals such as OH• can be effectively generated in air purged water under 185 nm VUV irradiation as given in equations (1.1)−(1.6). OH• radicals, with an oxidation–reduction standard potential of 2.80 V, are non-selective and vigorous oxidants. The rate constants for OH• reacting with most organic compounds are within the range of 10^6^−10^9^ l/(mol s) [43]. Therefore, under UV_185+254 nm_ irradiation, two degradation mechanisms as shown in figure 7, i.e. direct UV_254 nm_ photolysis and indirect oxidation with free radicals such as OH•, should be responsible for the decomposition of collectors [44].

Nevertheless, the contributions of UV_254 nm_ photolysis and indirect oxidation with free radicals to collector decomposition were quite dependent on molecular structures of thiol collectors. For the PEX, SDD and DDA, both UV_254 nm_ photolysis and indirect oxidation with free radicals had contributed greatly as the *k*_collector_ values under UV_185+254 nm_ irradiation were 1.60−2.63 times higher than those under UV_254 nm_ irradiation. But for the IET, the *k*_IET_ for UV_185+254 nm_ photolysis was 10.03 times higher than that for UV_254 nm_ photolysis. This suggested that indirect oxidation with free radicals was the main mechanism for the UV_185+254 nm_ photolysis of IET.

## 4. Conclusion

Thiol collectors (PEX, SDD, IET and DDA) could be effectively degraded by the UV_185+254 nm_ photolysis without dosing any oxidant. The removal efficiencies of PEX, SDD and IET reached nearly 100% upon 75 min of UV_185+254 nm_ irradiation. The *k*_collector_ for four collectors decreased in the order *k*_SDD_ > *k*_PEX_ > *k*_IET_ ≫ *k*_DDA_. The DDA was the typical refractory flotation collector for UV_185+254 nm_ photolysis. In the UV_185+254 nm_ photolysis of the PEX and DDA, the *k*_collector_ values were decreased at high initial pH, indicating neutral pH of flotation wastewaters can facilitate the collector decomposition. After 120 min of UV_185+254 nm_ irradiation, the *γ*_c_ and *γ*_s_ for four collectors reached 15−45% and 25−75%, respectively, with the mineralization extent of PEX > SDD > IET > DDA. The effective degradation of thiol collectors was attained under UV_254 nm_ irradiation alone. Thus, two mechanisms, i.e. direct UV_254 nm_ photolysis and indirect oxidation with free radicals such as OH•, were responsible for the decomposition of collectors by the UV_185+254 nm_ photolysis.

After UV_185+254 nm_ irradiation for 120 min, the percentage of gaseous sulfur was 1.57% for PEX, 4.85% for SDD, 0.53% for IET and 0.48% for DDA, respectively, indicating that only a small fraction of sulfur in collectors was released into emission gas. For each collector, the amount of emitted CS_2_ in gas phase was larger than that of H_2_S. The aqueous CS_2_ concentration increased rapidly in the first 10−20 min for each collector, and was then reduced to a low level of 0.05−0.1 mg l^−1^ at 120 min under UV_185+254 nm_ irradiation.

## Supplementary Material

Supplementary Material

Reviewer comments

## Data accessibility

The datasets supporting this article have been uploaded as part of the electronic supplementary material.
