# Supplementary material for: UV185+254 nm photolysis of typical thiol collectors: decomposition efficiency, mineralization and formation of sulfur byproducts
Source: R Soc Open Sci. 2019 May 22;6(5):190123. doi: 10.1098/rsos.190123 (PMC6549947; doi:10.1098/rsos.190123)
Supplement: Supplementary Material [file rsos190123supp1.docx]

**Electronic Supplementary Material from:**

**UV_185+254nm_ photolysis of typical thiol collectors: Decomposition efficiency, mineralization and formation of sulfur byproducts**

Pingfeng Fu^*^, Gen Li, Xiaoting Wu, Xiaofeng Lin, Bolan Lei

*School of Civil and Resources Engineering, University of Science and Technology Beijing, Beijing 100083, China.*

**Table S1** The variations of ln(*C*/*C*_0_) versus time *t* for UV_254nm_ photolysis of PEX, SDD, IET and DDA

| PEX | | SDD | | IET | | DDA | |
| --- | --- | --- | --- | --- | --- | --- | --- |
| time (min) | ln(*C*/*C*_0_) | time (min) | ln(*C*/*C*_0_) | time (min) | ln(*C*/*C*_0_) | time (min) | ln(*C*/*C*_0_) |
| 0 | 0 | 0 | 0 | 0 | 0 | 0 | 0 |
| 2 | -0.09524 | 2 | -0.12541 | 5 | -0.01645 | 10 | -0.07634 |
| 4 | -0.18604 | 4 | -0.27163 | 10 | -0.02854 | 20 | -0.12001 |
| 6 | -0.32975 | 6 | -0.48588 | 15 | -0.05413 | 40 | -0.22627 |
| 8 | -0.41326 | 8 | -0.81471 | 20 | -0.06378 | 60 | -0.28688 |
| 10 | -0.50254 | 10 | -1.03283 | 30 | -0.12237 | 80 | -0.34493 |
| 15 | -0.92621 | 15 | -1.6858 | 45 | -0.20454 | 100 | -0.42116 |
| 20 | -1.37709 | 20 | -2.22689 | 60 | -0.28684 | 120 | -0.50626 |

**Table S2** The variations of ln(*C*/*C*_0_) versus time *t* for UV_185+254nm_ photolysis of PEX, SDD, IET and DDA

| PEX | | SDD | | IET | | DDA | |
| --- | --- | --- | --- | --- | --- | --- | --- |
| time (min) | ln(*C*/*C*_0_) | time (min) | ln(*C*/*C*_0_) | time (min) | ln(*C*/*C*_0_) | time (min) | ln(*C*/*C*_0_) |
| 0 | 0 | 0 | 0 | 0 | 0 | 0 | 0 |
| 2 | -0.14699 | 2 | -0.33772 | 5 | -0.15683 | 10 | -0.0574 |
| 4 | -0.29614 | 4 | -0.56975 | 10 | -0.25633 | 20 | -0.15053 |
| 6 | -0.75334 | 6 | -0.83778 | 15 | -0.42317 | 40 | -0.29256 |
| 8 | -0.9343 | 8 | -1.42681 | 20 | -0.74666 | 60 | -0.46412 |
| 10 | -1.54284 | 10 | -1.8754 | 30 | -1.19675 | 80 | -0.56675 |
| 15 | -2.42063 | 15 | -3.1905 | 45 | -2.15001 | 100 | -0.69645 |
| 20 | -3.35501 | 20 | -4.16993 | 60 | -2.81614 | 120 | -0.81381 |

**Table S3** The variations of ln(*C*/*C*_0_) versus time *t* for UV_185+254nm_ photolysis

of PEX at initial pH 7‒12

| time *t* (time) | ln(*C*/*C*_0_) | | | | |
| --- | --- | --- | --- | --- | --- |
|  | pH=7.0 | pH=9.0 | pH=10.0 | pH=11.0 | pH=12.0 |
| 0 | 0 | 0 | 0 | 0 | 0 |
| 2 | -0.15979 | -0.15316 | -0.14699 | -0.09413 | -0.07267 |
| 4 | -0.38967 | -0.3937 | -0.29614 | -0.2297 | -0.18167 |
| 6 | -1.06883 | -0.91204 | -0.75334 | -0.60008 | -0.47435 |
| 8 | -1.49838 | -1.13795 | -0.9343 | -0.80116 | -0.65948 |
| 10 | -2.25466 | -1.80504 | -1.54284 | -1.13608 | -0.9465 |
| 15 | -3.76488 | -2.92282 | -2.42063 | -1.90704 | -1.55886 |
| 20 | -4.83825 | -4.10601 | -3.35501 | -2.61072 | -2.09851 |

**Table S4** The variations of ln(*C*/*C*_0_) versus time *t* for UV_185+254nm_ photolysis of

DDA at initial pH 7‒12

| time t (min) | ln(*C*/*C*_0_) | | | | |
| --- | --- | --- | --- | --- | --- |
|  | pH=7.0 | pH=9.0 | pH=10.0 | pH=11.0 | pH=12.0 |
| 0 | 0 | 0 | 0 | 0 | 0 |
| 10 | -0.14539 | -0.11935 | -0.0574 | -0.07559 | -0.05353 |
| 20 | -0.26596 | -0.19326 | -0.15053 | -0.13539 | -0.089 |
| 40 | -0.42259 | -0.35754 | -0.29256 | -0.24766 | -0.16211 |
| 60 | -0.53054 | -0.50823 | -0.46412 | -0.41498 | -0.2978 |
| 80 | -0.79775 | -0.66372 | -0.56675 | -0.49343 | -0.36229 |
| 100 | -0.92459 | -0.7921 | -0.69645 | -0.60953 | -0.50443 |
| 120 | -1.06988 | -0.914 | -0.81381 | -0.69461 | -0.59444 |

**Table S5** The variations of ln(*COD*/*COD*_0_) versus time *t* for UV_185+254nm_ photolysis of

PEX, SDD, IET and DDA

| PEX | | SDD | | IET | | DDA | |
| --- | --- | --- | --- | --- | --- | --- | --- |
| time (min) | ln(*COD*/*COD*_0_) | time (min) | ln(*COD*/*COD*_0_) | time (min) | ln(*COD*/*COD*_0_) | time (min) | ln(*COD*/*COD*_0_) |
| 0 | 0 | 0 | 0 | 0 | 0 | 0 | 0 |
| 4 | -0.06758 | 4 | -0.07 | 10 | -0.03535 | 20 | -0.09259 |
| 10 | -0.24155 | 15 | -0.17008 | 20 | -0.11298 | 40 | -0.13228 |
| 20 | -0.47344 | 20 | -0.23739 | 30 | -0.15759 | 60 | -0.18486 |
| 30 | -0.76469 | 30 | -0.33004 | 45 | -0.24577 | 80 | -0.22223 |
| 45 | -0.93676 | 45 | -0.4375 | 60 | -0.30478 | 100 | -0.24006 |
|  |  | 60 | -0.51827 | 90 | -0.42558 | 120 | -0.28467 |
|  |  |  |  | 120 | -0.495 |  |  |
